# What the oncologist needs to know about COVID-19 infection in cancer patients

**DOI:** 10.2217/fon-2020-0312

**Published:** 2020-04-23

**Authors:** Elie Rassy, Rita-Maria Khoury-Abboud, Nathalie Ibrahim, Clarisse Kattan, Tarek Assi, Joseph Kattan

**Affiliations:** 1 Department of Medical Oncology, Hotel Dieu de France University Hospital, Faculty of Medicine, Saint Joseph University, Lebanon

**Keywords:** chemotherapy, coronavirus, COVID-19, immune checkpoint inhibitors, oncologist, oncology, review

The outbreak of Coronavirus Disease 2019 (COVID-19) started back in December 2019 with a cluster of pneumonia cases in Wuhan, a city within the central part of China, and has rapidly evolved into a global pandemic [1]. The putative pathogen is a novel coronavirus that presents a close resemblance to a known bat coronavirus termed BatCoV RaTG13, thus favoring a bat-to-human transmission hypothesis before eventually identifying human-to-human transmission [2,3]. It presents a high transmission rate with one new infected case producing an average of 2.9 new secondary cases and a fatality rate of 2.3% [4,5]. As a result, thousands of severe cases have died every day worldwide due to the pressure on the healthcare system and lack of specific treatments. Not surprisingly, cancer patients, compared with the general population, are regarded as a highly vulnerable group because of their immunosuppressive state due to malignancy, chemotherapy and comorbidities. Thus, oncologists are obliged to reconsider anticancer treatments while taking into consideration the risk of complication and cancer progression [6]. An updated WHO report demonstrates a mortality of 7.6% among patients with cancer [7]. Subsequently, several societies have issued conservative guidelines inviting oncologists to consider, on a case-by-case basis, the possibility of delaying treatment administration [8–12]. In this paper, we provide the oncologists with the available evidence concerning COVID-19 in cancer patients to better guide management decisions.

## COVID-19 infections in cancer patients

As China was the first epicenter for the pandemic, the Chinese epidemiology data constitute the bulk of the published literature reporting on COVID-19 infections. The early case series including a total of 300 COVID-19 patients identified 2 cancer patients only [4,13]. Later case series by Liang *et al.* (18 cases), Zhang *et al.* (28 cases) and Zhang *et al.* (67 cases) reported a higher prevalence of cancer patients with COVID-19 infections compared with the overall population (1 vs 0.29%) [14], a higher median age at diagnosis (63–66 vs 49 years) [14–16] and a male predominance (61%) [15,16]. Lung cancers (22–25%), gastrointestinal cancers (14–16%) and breast cancers (11%) were the most commonly encountered tumors [15,16]. The clinical features included fever (80–82%), dry cough (75–81%) and dyspnea (50–66%) [15,16]. Dyspnea was more frequently noted at admission in severe cases (56.3 vs 11.4%) and in nonsurvivors (66.7 vs 20.4%) whereas the other symptoms were similar between mild and severe cases [16]. Laboratory tests showed hypoproteinemia (89%), lymphopenia (82%), increased level of CRP (82%) and anemia (75%) [15]. In comparison with patients without cancer, cancer patients had a higher risk of adverse events (39 vs 8%; p = 0.0003) and deteriorated more rapidly (13 vs 43 days, HR = 3.56; 95% CI: 1.65–7.69) [14]. Severe events were reported in 48–54% of cases (versus 16% in the overall population), notably among patients receiving anticancer treatment within the previous 2 weeks (OR = 4.079; 95% CI: 1.086–15.322) [14–16]. Compared with the mild illness group, patients in the severe illness group were older (69 vs 64 years; p < 0.001) and had more comorbidities (72 vs 37%; p = 0.004) [16]. Serious complications included acute respiratory distress syndrome (20.9 vs 3.4% in the overall population), heart failure (16.4%) and acute renal injury (3 vs 0.5% in the overall population) [16,17]. Empirical antibiotics, antiviral agents, glucocorticoids and intravenous immunoglobulins were administered in 82, 71–85, 45 and 20–26%, respectively [15,16]. Oxygen therapy, noninvasive ventilation and invasive mechanical intubation were required in 73, 30 and 12–36%, respectively [14–16]. Cancer patients had a higher case-fatality rate (5.6–29 vs 1% in the overall population) [14–16]. The median duration to recovery and death was 31 and 20 days, respectively [16].

## Diagnosis of COVID-19 infections

The diagnosis of COVID-19 seems obvious but is not straightforward in clinical practice. Patients may be very symptomatic at presentation showing fever and respiratory symptoms, which are very commonly encountered in daily practice. The COVID-19 diagnosis adds to a long list of differential diagnoses including bacterial, fungal or other viral infections. Patients may also present with very subtle symptoms that may not be clinically relevant. For example, the earliest reports from Wuhan described two patients presenting ground-glass opacities in their lungs, a characteristic radiological finding in COVID-19 patients, who had undergone lobectomies to remove early-stage lung cancers but ended up having a COVID-19 infection. Both patients eventually became severely ill, and one of them died of respiratory failure [18]. COVID-19 also adds to the etiologies of pneumonitis following cytotoxic chemotherapies, immune checkpoint inhibitors and radiotherapy. In such instances, steroids are the mainstay of any treatment plan however its use during COVID-19 infection is controversial as it slows the elimination of the virus. The confirmation of a COVID-19 infection is currently largely based on reverse-transcriptase polymerase chaine reaction (RT-PCR). This technique requires a deep nasopharyngeal swab sampling and is available broadly. However, RT-PCR testing seems to present low accuracy especially in places that perform large numbers of tests. In one case series of 1014 patients, 75% of patients with negative RT-PCR had positive chest computed tomography findings of COVID-19 infections (48% highly likely cases and 33% probable cases) and were attributed to faulty design of some PCR kits and inadequate sampling [19].

## Anticancer treatment during COVID-19 infections

Most patients with cancer were recommended to withdraw or delay cancer treatment during the pandemic as almost 30% of cancer patients’ infection was suspected to be hospital-associated transmission [15]. However, the risks of cancer progression make this issue controversial. In contrast to chemotherapy which is immunosuppressive, immune checkpoint inhibitors may be a safer option as one case series of cancer patients with COVID-19 infection did not report any case receiving immunotherapy [14]. Thus, patients may be less prone to severe infections but are at a theoretical risk of a cytokine release syndrome that would exacerbate a COVID-19 infection [20–22]. The biologic findings including lymphopenia, neutrophilia, elevated D-dimer and LDH very frequently encountered in cancer patients seem to increase the risk of severe COVID-19 infections [23]. A case report of a patient with EGFR (L858R, T790M) mutant metastatic lung adenocarcinoma and diagnosed with COVID-19 infection maintained his daily osimertinib concomitantly with broad-spectrum antibiotics and antiviral treatment with lopinavir plus ritonavir uneventfully [24]. Concerning clinical trials inclusions, the US FDA and the EMA have issued special guidance for the conduction of clinical trials during the COVID-19 pandemic [25,26].

Cancer patients with suspected or confirmed COVID-19 should be discussed with an infectious disease specialist. Based on the data suggesting patients with cancer are at high risk of respiratory complications related to COVID-19 infection, many societies favor delaying treatments on a case-by-case basis [8–12]. The treatment of COVID-19 has been a matter of controversy with one single-arm trial showing the potential efficacy of the azithromycin-hydroxychloroquine combination. Unfortunately, this study had major methodology issues and was not adopted by the medical society [27]. In the absence of solid evidence for effective antiviral therapy, the research activity has never been this active. The number of ongoing trials registered increased from 84 trials on 24 March (at the conception of the paper) to 306 on 4 April 2020 (at the time of submission). Several therapies varying from classical antiviral drugs such as lopinavir-ritonavir (NCT04330690 and NCT04307693 currently recruiting, NCT04321993 active but not yet recruiting) and remdesivir to unconventional treatments such as chloroquine and hydroxychloroquine (NCT04328272 and NCT04307693 currently recruiting, NCT04321993 active but not yet recruiting) are undergoing evaluation in randomized clinical trials. The role of immune therapies is also being explored in patients with severe infections including, tocilizumab an anticytokine therapy which binds IL-6 receptors (NCT04317092 currently recruiting), hyperimmune plasma (NCT04321421 active but not yet recruiting). The eagerly awaited study is the Phase III trial (DisCoVeRy, NCT04315948) randomizing 3100 patients to remdesivir, lopinavir-ritonavir, IFNβ-1A, hydroxychloroquine and standard of care.

## Conclusion & perspective

At present, there is a global pandemic of COVID-19 that has infected more than 1 million cases and killed more than 60,000 cases [28]. In comparison with the overall population, cancer patients are at a higher risk of severe events in 48–54% of cases (vs 16% in the overall population) and death in 5.6–29% (vs 3.4% in the overall population on 3 March 2020 vs 2% in the overall population on 10 February 2020) [28]. The current evidence remains insufficient to explain a conclusive association between cancer and COVID-19. The majority of the position papers and guidelines were based on the epidemiology data of Liang *et al.* published on 1 March 2020 [8–12,14]. However, 12 of the 18 cancer patients reported by Liang *et al.* were older than the general population, had no active cancer and were long-term cancer survivors [14]. The other case series do not circumvent this issue as Zhang *et al.* reported a concomitant chronic disease in 64% of cancer patients and higher fatality rate among patients in the active treatment phase in comparison with those at the follow-up phase (39 vs 21%) [16]. The relatively small sample size, limited clinical information and heterogeneity of the disease course between patients limit robust conclusions. At last, the higher rate of cancer patients with COVID-19 could be biased and related to the closer medical follow-up of these patients and the higher mortality to delayed hospitalization while coping with the rapid influx of severe cases. Several questions remain unanswered notably the risks of waiting for the COVID-19 epidemic to subside before treating cancer patients or the risks of exposure to this virus during admission for cancer treatment. This risk should be particularly assessed in patients that may be cured by oncologic treatments. Moreover, the risk of patients receiving hormonal therapy, immune checkpoint inhibitors and targeted therapies should be assessed. Today, abiding by the old *primum non nocere* concept, clinicians may have to balance the risks of developing a COVID-19 infection against the risks of tumor progression, while taking into consideration the prevailing state of the healthcare system.
